# Cervical cancer testing among women aged 30–49 years in the WHO European Region

**DOI:** 10.1093/eurpub/ckab100

**Published:** 2021-09-07

**Authors:** Julianne Williams, Ivo Rakovac, Jocelyn Victoria, Tatiana Tatarinova, Marilys Corbex, Ben Barr, Tanith Rose, Lela Sturua, Galina Obreja, Diana Andreasyan, Shukhrat Shukurov, Hagverdiyev Gahraman, Bente Mikkelsen, Nino Berdzuli, João Breda

**Affiliations:** 1 The World Health Organization European Office for the Prevention and Control of Noncommunicable Diseases, Division of Country Health Programmes, World Health Organization Regional Office for Europe, Moscow, Russian Federation; 2 Institute for Leadership and Health Care Management, I.M. Sechenov First Moscow State Medical University (Sechenov University), Moscow, Russian Federation; 3 Division of Country Health Programmes, World Health Organization Regional Office for Europe, Copenhagen, Denmark; 4 Department of Public Health and Policy, Institute of Population Health Sciences, University of Liverpool, Liverpool, UK; 5 Noncommunicable Disease Department, National Center for Disease Control and Public Health, Tbilisi, Georgia; 6 Department of Social Medicine and Health Management, State University of Medicine and Pharmacy, Chisinau, Republic of Moldova; 7 Department of National Health Information Analytic Center, National Institute of Health, Yerevan, Republic of Armenia; 8 Central Project Implementation Bureau of the “Health-3” Project of the Ministry of Health and the World Bank, Tashkent, The Republic of Uzbekistan; 9 Public Health and Reforms Center, Ministry of Health of Azerbaijan Republic, Baku, Azerbaijan

## Abstract

**Background:**

Screening programs play an important role in a comprehensive strategy to prevent cervical cancer, a leading cause of death among women of reproductive age. Unfortunately, there is a dearth of information about rates of cervical cancer testing, particularly in Eastern Europe and Central Asia where levels of cervical cancer are among the highest in the WHO European Region. The purpose of this article is to report on the lifetime prevalence of cervical cancer testing among females aged 30–49 years from across the WHO European region, and to describe high-level geographic and socioeconomic differences.

**Methods:**

We used data from the European Health Information Survey and the WHO STEPwise approach to Surveillance survey to calculate the proportions of women who were tested for cervical cancer.

**Results:**

The percentage of tested women ranged from 11.7% in Azerbaijan to 98.4% in Finland, with the lowest percentages observed in Azerbaijan, Tajikistan and Uzbekistan. Testing was lower in Eastern Europe (compared to Western Europe), among low-income countries and among women with lower levels of education.

**Conclusion:**

Effective cervical cancer screening programs are one part of a larger strategy, which must also include national scale-up of human papilloma virus vaccination, screening and treatment.

## Introduction

Cervical cancer is a leading cause of death among women of reproductive age.^1–3^ It often occurs during women’s most productive years, posing tremendous human and economic costs as well as threatening sustainable development. Cervical cancer is primarily caused by persistent or chronic infection with human papilloma virus (HPV), a sexually transmitted infection usually acquired early in sexual life.^4^ While most HPV infections clear up on their own (more than 90% of new HPV infections at any age regress in 6–18 months^5^^,^^6^) for some women it progresses to invasive cervical cancer. Screening programmes, which identify asymptomatic diseases in an apparently healthy target population^7^^,^^8^ play an important role in early detection and treatment of precancerous lesions and the reduction of incidence and mortality from cervical cancer.^9^ Because cervical cancer is both preventable and curable if diagnosed early and treated effectively, it is possible to eliminate it and significantly reduce premature mortality.^10^^,^^11^ Accordingly, cervical cancer screening has been identified as a cost-effective intervention for preventing non-communicable diseases (NCDs)^12^ and is one indicator within the WHO Global Monitoring Framework for NCDs.

**Figure 1:**
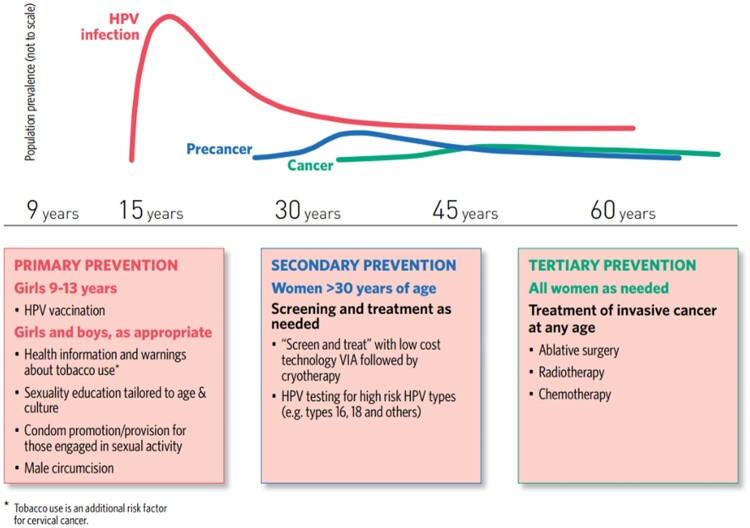
WHO comprehensive approach to cervical cancer prevention and control: conceptual description of the primary, secondary and tertiary prevention of cervical cancer applied throughout life-course^14^

Cervical cancer control requires a comprehensive approach,^13^ including primary, secondary and tertiary prevention^14–16^ (figure 1). Following this approach, the WHO has developed a strategy for cervical cancer elimination which requires national scale-up of HPV vaccination, screening and treatment. It proposes that by 2030, 90% of girls be fully vaccinated against HPV by age 15, 70% of women be screened with a high-performance test by age 35 (and again by age 45) and 90% of women with cervical disease receive treatment (90% of women with pre-cancer treated; 90% of women with invasive cancer managed).^14^

In order to monitor progress in addressing cervical cancer, the WHO recommends the following national performance indicators: HPV vaccination coverage, disaggregated by age at vaccination and number of doses; screening rate of the target population (women 30–49 years); percentage of women aged 30–49 who have been screened for the first time in the past 12 months; and percentage of screened women aged 30–49 years with a positive screening test result in the last 12 months.^17^ The WHO Global Monitoring Framework on Non-communicable Diseases also includes an indicator representing ‘the proportion of women between ages of 30–49 screened for cervical cancer at least once, or more often, and for lower or higher age groups according to national programmes or policies’.^18^

This article focuses on one of the early steps in identifying and treating women with precancerous lesions: the process of testing for cervical cancer. Within the WHO European Region, cervical cancer screening policies and coverage vary widely. This variation mirrors large national differences in cervical cancer incidence and mortality, which are generally higher in the East than elsewhere in the region.^1^^,^^19^ It also corresponds with important differences in the implementation of screening programs, which have been described as being either ‘organized’ (proactive, structured, and well-targeted) or ‘opportunistic’ (reactive, on-request and less systematic).^20^ Evidence suggests that organized programs are more effective and less costly than opportunistic approaches.^21–23^

The process of testing for cervical cancer (which we will refer to here as ‘smear testing’ or simply ‘testing’) may refer to a number of different testing methodologies, including cytology tests (e.g. Papanicolaou, Pappenheim, Romanowsky–Giemsa) and molecular HPV screening tests. Providing these tests to the largest possible proportion of relevant women is the start of the cervical cancer screening process,^4^ and is normally followed by ongoing management when the test result is positive.

Despite the inclusion of indicators for cervical cancer screening in the Global Monitoring Framework^18^ and WHO recommendations,^4^ there is a dearth of publicly available information about screening practices, particularly in Eastern Europe and Central Asia. In view of this research gap, this article aims to provide baseline data about lifetime cervical cancer testing levels (among females aged 30–49) for a large number of countries. It will also examine socioeconomic differences within countries, as previous research has shown women from low socioeconomic groups are less likely to undergo a pelvic exam or pap smear, potentially increasing their risk of having cervical cancer later in life.^24^^,^^25^ Using harmonized indicators and nationally representative samples, this article aims to improve understanding about differences in testing rates and inequalities, both between and within countries. This knowledge will ultimately enhance monitoring, research and implementation efforts in the global effort to eliminate cervical cancer.^14^

## Methods

### Data sources and measures

We used data from two main sources. For European Union countries (along with Iceland and Norway), our source was the European Health Interview Survey (EHIS). The EHIS is a general population survey (of people aged 15 or over living in private households), which asks questions about health status, health determinants and healthcare activities. We used data from wave 2 of EHIS, conducted between 2013 and 2015. One of its questions asked women, ‘When was the last time you had a cervical smear test?’ Possible answers were (i) ‘Within the past 12 months’; (ii) ‘1 to less than 2 years’; (iii) ‘2 to less than 3 years’; (iv) ‘3 years or more’ and (v) ‘Never’. These responses were recoded into a binary variable representing whether the respondent had ever—or never—had a cervical smear test. (EHIS methods are provided in detail elsewhere.^26–28^)

For all other countries, we used data from the WHO STEPwise approach to Surveillance (STEPS) surveys. The STEPS surveys represent one of the most internationally comparable and integrated data sources available about NCDs and their risk factors.^29^ The STEPS surveys were carried out between 2013 and 2017 in Armenia, Azerbaijan, Georgia, Kyrgyzstan, Tajikistan, the Republic of Moldova, Uzbekistan, Romania, Turkey and Turkmenistan. (Main findings from these studies are available on the WHO website.^30^) STEPS employed multi-staged cluster sampling to draw a nationally representative sample of adults aged 18–69 years, then delivered the survey via trained interviewers using a standard protocol detailed elsewhere.^31^ When asking female participants about cervical cancer testing, STEPS interviewers prefaced their questions with background information about the topic. Specifically, the language used was ‘The next question is about cervical cancer prevention. Screening tests for cervical cancer prevention can be done in different ways, including visual inspection with acetic acid (VIA), Pap smear and human papillomavirus (HPV) test. VIA is an inspection of the surface of the uterine cervix after acetic acid (or vinegar) has been applied to it. For both a Pap smear and an HPV test, a doctor or nurse uses a swab to wipe from inside your vagina, take a sample and send it to a laboratory. It is possible that you were given the swab yourself and asked to swab the inside of your vagina. The laboratory checks for abnormal cell changes if a Pap smear is carried out and for HPV if an HPV test is carried out’. After providing this prefatory context, the interviewers then asked: ‘Have you ever had a screening test for cervical cancer, using any of these methods described above?’ Possible answers were ‘yes,’ ‘no’ and ‘don’t know.’ (For Turkey, the question was different, asking: ‘When did you last have a cervical smear test?’ Response options were ‘Last 12 months’, ‘>1 to <2 years’, ‘>2 to <5 years’, ‘>5 years’, and ‘never’.) These options were recoded to form a binary variable consisting of ‘never’ vs. all other responses.

Education categories were mapped to three broad groups. In the EHIS, participants were asked about their highest level of education completed. We recoded their response options into a three-category variable: (i) low education—pre-primary to lower secondary education only, (ii) medium education—upper secondary to post-secondary non-tertiary education and (iii) high education—tertiary education. Similarly, for STEPS data we created a three-category education level variable using information on years spent at school or in full-time study (excluding pre-school), with 0–10, 11–12 and 13+ years of education denoting the categories.

Countries were grouped into four income categories according to World Bank criteria: high, upper-middle, lower-middle and low. This income classification is based on a measure of national income per person, or gross national income per capita, calculated using the Atlas method.^32^

Ethics approval was obtained from the relevant authorities in each country and all participants provided informed consent.^26^ EHIS data were obtained through a data sharing agreement with Eurostat (agreement RPP 85/2018-LFS-EU-SILC-EHIS).

### Statistical analysis

Only women aged 30–49 years were included in our analysis. This is the age range used in the WHO’s cervical cancer screening recommendation,^4^ as well as in indicator 25 of the Global Monitoring Framework which monitors cervical cancer screening.^18^ We calculated point estimates for the percentage of women aged 30–49 years who reported ever having had a cervical cancer screening, along with corresponding 95% confidence intervals, by country and education level. The denominator of this percentage was surveyed women aged 30–49 years, and the numerator was women aged 30–49 years who reported ever having had a cervical cancer screening test.

Data were analysed using an R package for statistical computing using appropriate sampling weights to ensure representativeness at the national level and adjusted for national age distribution, clustered sampling methods and non-response rate. (Reports of sample sizes and response rates for individual countries are available on the EHIS and STEPS websites.)

## Results

**Figure 2:**
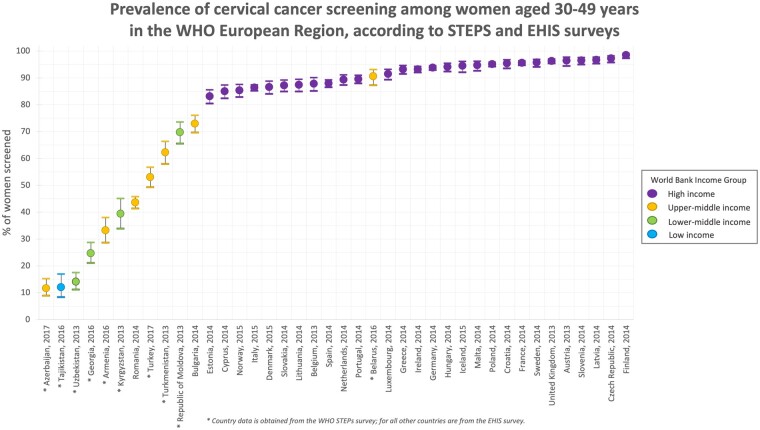
Proportion of women aged 30–49 years who reported receiving a cervical smear test, by country and year

The proportion of women aged 30–49 years who reported ever having had a cervical cancer test ranged widely across the European region, from 11.7% in Azerbaijan to 98.4% in Finland. The lowest levels were seen in Azerbaijan, Tajikistan and Uzbekistan (figure 2). Clear geographical patterns emerged, with higher levels across Western Europe and lower levels in Eastern Europe.

There were also sizable differences according to country income (figure 2). In high-income countries, testing levels were high and variance was modest: Finland led with 98.5%, and even the lowest country, Estonia, reported 83.1%. However, in upper-middle-income countries (Armenia, Azerbaijan, Belarus, Bulgaria, Romania, Turkey and Turkmenistan), the differences were much more pronounced. Testing rates in those countries ranged from 11.7% in Azerbaijan to 90.6% in Belarus. Similarly, lower-middle-income countries (Georgia, Kyrgyzstan, Republic of Moldova and Uzbekistan) had wide variation, from 14.05% in Uzbekistan to 69.7% in the Republic of Moldova. (Only one low-income country was included in the data: Tajikistan, which had a testing rate of 11.99%, the second-lowest in the sample.) Although testing rates tended to increase with the income of a country, there were some exceptions. For example, Belarus had testing rates of 90.6%, which were comparable to the rates seen in high-income countries, despite being an upper-middle-income country. The Republic of Moldova also had much higher testing rates compared to other lower-middle-income countries.

**Figure 3:**
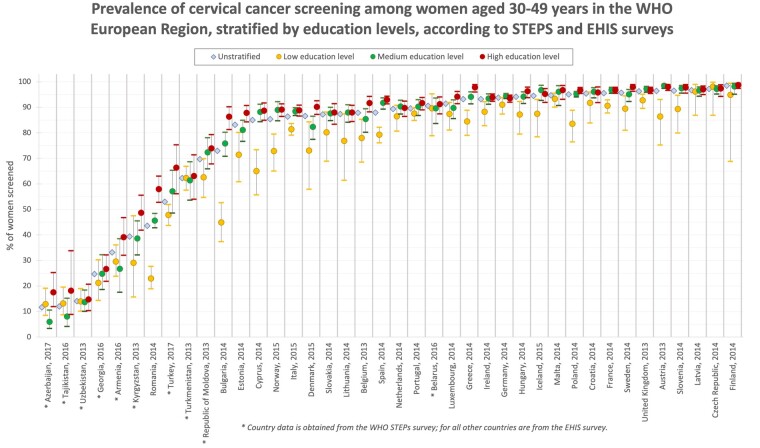
Proportion of women aged 30–49 years who reported receiving a cervical smear test, stratified by education levels (low, medium and high), by country and year

Distinct differences also emerged according to education level: higher rates were found in women with higher education and lower in women with lower education (figure 3). This was the case for the majority of countries, with five exceptions: Uzbekistan, Turkmenistan, Belarus, Latvia and Czech Republic. In those countries, the proportion of women who reported being tested was virtually the same across education levels. The lowest levels of testing among education level/country subgroups were found in Azerbaijan (5.98% among medium education level) and Tajikistan, while the highest levels were found in the Czech Republic (98.1% for low education level), Austria (98.4% for medium education level) and Finland (98.7% for high education level).

## Discussion

To the best of our knowledge, this is the first large multi-country publication of lifetime prevalence of cervical cancer smear testing among women aged 30–49 years in Europe and Central Asia. It also brings data into the international literature about testing in Eastern Europe and Central Asia, which has been less readily available, despite its importance as a key indicator in the WHO Global Monitoring Framework.^18^

To be sure, the data here reveal few surprises. The landscape of cervical cancer screening across Europe and Central Asia is already understood as one of high variation across national, geographic and socioeconomic lines. This is to be expected in light of historical trends, such as the steady rise in incidence of cervical cancer per 100 000 women in Eastern European and Central Asian countries since the 1990s.^33^ It is also to be expected given the frequent constraints on resources, political motivation and operational clarity that lower-income countries face, which can make it difficult to ensure the provision of adequate cancer services. Additionally, the data here show that high variation exists in testing rates between education levels, which also broadly aligns with previous research.^24^^,^^34^

It is important to note, however, that even countries with strong performance on these indicators may still have substantial progress to make. High coverage of testing at the national level does not necessarily entail widespread provision of ‘effective’ or ‘high-quality’ screening. For example, in some countries (e.g. Belarus and Republic of Moldova), there is high coverage of reported smear testing, but also high incidence of cervical cancer.^35^ This suggests that the quality of the programmes (rather than only the amount of testing) is a key issue for future consideration. For example, the Romanowsky–Giemsa staining, which is not recommended by the WHO,^4^ continues to be used in some Eastern European and Central Asian countries,^36^ along with opportunistic annual cytology screening practices among broad age groups. Experts advocate for a shift in policy away from these opportunistic screenings and towards population-based, quality-assured HPV vaccination and HPV-based screening programmes.^37^

### Limitations and future research

This study inherited a number of limitations from EHIS and STEPS, two different surveys that use slightly different methods and questions. In some countries, response rates were low.^38^ Additionally, response bias may have been introduced via recall bias, societal or cultural acceptance, uncertainty about the performed test or procedure, non-response bias and lower inclusion of women from marginalized and disadvantaged socioeconomic groups. For an extended discussion of the limitations, please see Supplementary file S1.

There are a number of questions that would have been interesting to ask, but impossible to answer with this data. One such question is how many tests a woman has ever received (as opposed to whether or not she has ever received any). Experts on WHO missions have noticed over-screening (testing yearly, sometimes up to five times yearly) in many Eastern European and Central Asian countries, where the perceived necessity of annual testing persists among health providers and women. Yet over-testing can lead to many other problems—including false positives/negatives, psychosocial concerns, over-diagnosis/treatment and wasted resources—so it would be valuable for future research to measure this.^7^^,^^20^ Another interesting yet infeasible question would have involved comparing countries based on characteristics of their healthcare systems, for instance whether screenings are ‘organized’ or ‘opportunistic’ in nature. However, this information is currently being collected in the WHO Country Capacity Survey for the Prevention and Control of Non-communicable Diseases, which can inform future research.^39^

Several other questions would have been interesting to ask but were simply beyond the scope of this analysis. We did not differentiate between the types of testing received (many survey participants may be unfamiliar with methods of sample analysis, and countries may provide different types of staining). Nor did we consider follow-up actions that take place after screenings occur. While effective cervical cancer prevention relies on following positive test results with effective treatment (the ‘screen-and-treat’ or ‘screen, diagnose and treat’ approach), our analysis was focused narrowly on the question of testing only.^4^ Similarly, we chose not to address the role of HPV vaccination. Availability of total or partial charge HPV vaccination varies across Europe, with higher levels of immunization in higher-income countries in Western Europe.^40^ Currently, only a few Eastern European and Central Asian countries have HPV vaccination programmes,^41^ and they were introduced recently (please see Supplementary file S2 for all references beyond reference 40). Of course, while it is too early to examine generational effects in those countries, HPV vaccination remains a core component of the WHO Global Strategy for cervical cancer elimination.^14^

Additionally, we did not consider the woman’s age at the time of testing—only at the time of the survey (age 30–49). We chose this age range because WHO recommends that every woman in this age group be screened at least once,^4^ and also because this age range aligns with the one used in the Global Monitoring Framework for NCDs.^18^ However, the language of the WHO’s recommended performance indicator (‘percentage of women aged 30–49 years who report ever having had a cervical cancer test’^18^) leaves open the possibility that some women who reported having had a test received it prior to age 30, and not after. This possibility—as well as the WHO’s recommendation that 70% of women be screened with a high-performance test by age 35 and again by age 45^14^—means that having an indicator about number of tests and age at each test would be valuable. A refinement of the cervical cancer screening indicator is underway,^42^ and future studies may benefit on this point from the revised indicators.

### Policy implications

In the 1960s and 1970s, high-income countries had cervical cancer incidence and mortality rates that were similarly high to those we see in the developing world today. In the years since, the decline in those rates has largely been credited to effective screening programmes and treatment of precancerous lesions.^34^ Currently, cervical cancer estimates in several countries in the European region—including Kazakhstan, Kyrgyzstan, Republic of Moldova, the Russian Federation and Ukraine—are four or five times the threshold incidence rate^43^ set by the WHO Draft Global Strategy.^14^ These trends suggest a need for greater policy attention to prevention, early diagnosis and treatment. They also emphasize the importance of removing structural health care barriers that may influence women’s poor presentation for screening, such as inadequate health literacy or the lack of patient-centred health services.^44^

In all these areas, making progress depends on measuring progress. Having effective indicators is essential for setting and reaching strategic targets. Key programme indicators must include primary, secondary and tertiary prevention efforts, such as HPV vaccination, screening and treatment of pre-cancers, treatment of cancers and palliative care.^9^ They should provide visibility into screening programs’ quality and effectiveness, such as the principles for organized, population-based programs set out in the European Guidelines for Quality Assurance in Cervical Cancer Screening.^44,45^ As one potential starting point, a national cancer registry may help a country monitor long-term trends in incidence and mortality rates.

## Conclusions

In conclusion, this article establishes the most recent data for lifetime cervical cancer smear testing levels for women aged 30–49 years across the WHO European Region. Findings indicate that levels of testing are lower in Eastern Europe and Central Asian countries, and also among lower-income countries. We found that nearly all participating countries need to improve their reach among women of lower socioeconomic status. These findings can be useful to advocate for better screening, particularly among low-income women and women living in Central Asia and Eastern Europe. We hope that this publication will generate additional research into the root causes behind problems such as over-testing, poor screening coverage and low levels of follow-up. This article examined testing levels of women, which is the first step in the screening process. Effective strategies towards cervical cancer elimination will also require coordinated implementation of population-based, quality-assured HPV vaccination programmes, screening programmes (with appropriate follow-up), quality management of invasive cervical cancer (including palliative care) and cancer registries.

## Supplementary data

Supplementary data are available at *EURPUB* online.

## Supplementary Material

ckab100_Supplementary_Data
