# Supplementary material for: Cervical cancer testing among women aged 30–49 years in the WHO European Region
Source: Eur J Public Health. 2021 Sep 7;31(4):884–9. doi: 10.1093/eurpub/ckab100 (PMC8514175; doi:10.1093/eurpub/ckab100)
Supplement: ckab100_Supplementary_Data [file ckab100_supplementary_data.zip › ckab100-suppl_data/ejph-2020-07-om-0880-File005.docx]

# Supplementary File

# DISCUSSION (Extended)

To the best of our knowledge, this is the first large multi-country publication reporting on the lifetime prevalence of cervical cancer smear testing among women aged 30 – 49 years in Europe and Central Asia. One of the main contributions that this paper makes is that it provides information from Eastern Europe and Central Asia, where data on levels of testing are less readily available in international literature. This is an important indicator of the health system’s response to noncommunicable diseases, upon which all WHO Member States have agreed to report as part of the Global Monitoring Framework(18).

Findings indicate that there is wide between-country variation in the levels of cervical cancer testing among women aged 30-49 years in the European region, ranging from 11.7% in Azerbaijan to 98.4% in Finland. Rates of testing were generally higher in Western Europe and lower in Eastern Europe. Previous research examined cervical cancer screening in 57 countries using World Health Survey statistics, but it did not include findings from Armenia, Azerbaijan, Georgia, Republic of Moldova, Turkmenistan, Tajikistan or Uzbekistan(33). This study found wide variation in the level of coverage across countries(33). In some countries, a majority of women have had pelvic exams in their lifetime (defined as ‘crude coverage’), but the rates of ‘effective coverage’ (i.e. the proportion of women who had an exam in the past three years accompanied by laboratory tests) were low.

Findings from this study corroborate the findings of earlier research(24) and indicate little progress since the early 2000s, suggesting that women from lower socioeconomic groups are still considerably less likely to have smear testing. This lack of testing may be due to a number of factors including lower awareness of the benefits of screening, lack of time(24), inappropriate design of smear testing programme, absence of equitable access to services or absence of enabling policy measures. Other issues may relate to whether or not screening is easily physically and financially accessible for all women(34). For example, women of low socioeconomic status who live in rural areas may have difficulty accessing services due to the cost of transport. In instances where a pathology may be found, cost of treatment (even in instances where health insurance covers 80 or 90% of the costs) may not be affordable (37,38).

This study indicates that in the WHO European region, cervical cancer smear testing rates tended to be lower in lower income countries. Globally, the vast majority of cervical cancers occur in low- and middle-income countries and according to socio-economic development(37). In the 1960s and 1970s, incidence rates in high-income countries were similarly high to the rates seen in the developing world today; the decline in cervical cancer incidence and mortality in high income countries is largely credited to effective screening programmes and treatment of precancerous lesions (33). Lower-income countries face resource constraints which make it difficult to ensure that adequate cancer services are available for the population. In addition to scarce human or financial resources, other barriers may include a lack of political motivation, or a lack of clarity on operational guidelines, roles and responsibilities.

The incidence of cervical cancer per 100,000 women has been rising in Eastern European and Central Asian countries steadily since the 1990s (38). National estimates of cervical cancer estimates in countries including Kazakhstan, Kyrgyzstan, Republic of Moldova, the Russian Federation and Ukraine are currently four or five times the threshold incidence rate(39) set by the WHO Draft Global Strategy(14). These trends suggest a need for greater policy attention to prevention, early diagnosis and treatment.

At the national level, high coverage of testing does not necessarily mean that there is widespread provision of *effective* or *high quality* screening. For example, in some countries (e.g. Belarus and Republic of Moldova), there is high coverage of reported smear testing, but also high incidence of cervical cancer(40). This suggests that quality of the programmes (rather than coverage of testing) is a key issue for future consideration. Furthermore, there are important issues about the methods used for assessing the samples. For example, the Romanovsky Giemsa staining, which is not recommended by the WHO(4), continues to be used in some Eastern European and Central Asian countries (41), along with opportunistic annual cytology screening practices among broad age groups. Experts advocate for a shift in policy, away from these opportunistic screenings towards population-based, quality-assured HPV vaccination and HPV-based screening programmes(42).

At the same time, experts have also called for additional efforts to address structural health care barriers which may influence women’s poor presentation for screening, such as inadequate health literacy, or lack of patient-centered health services (43)

It is important to monitor and assess progress and targets in national strategies related to cervical cancer screening. Key programme indicators must include primary, secondary and tertiary prevention of cervical cancer such as HPV vaccination coverage, screening and treatment of precancers, treatment of cancers and palliative care (9). The European Guidelines for Quality Assurance in Cervical Cancer Screening(44) define the principles for organized, population-based screening, and discuss key aspects of effective implementation of screening programs, including quality assurance. Important considerations include the breadth of screening coverage (including what proportion of the target population is reached), the duration of screening programs and the frequency in which they are implemented. It is recommended that intervals between screening should be three years or less(45). National cancer registries may help monitor long-term trends in disease incidence and mortality rates, to assess both the impact of HPV vaccination and cervical cancer screening and treatment programmes.

It is important to address some of the limitations of this analysis, including survey response rates that were low in some countries(46). The proportions of women reported to have been tested may be biased because of several factors, including recall bias, societal and cultural acceptance, uncertainty of the performed test or procedure, non-response bias and lower inclusion of women from marginalized and disadvantaged socio-economic groups in the survey. Furthermore, this survey combined data from two different surveys with slightly different methods and questions, and made comparisons in cervical cancer testing levels between countries with diverse health care systems and diverse methods of testing. Future work should investigate how the characteristics of the health care screening, including features such as the cost of screening or access to screening services may influence screening levels in the population. Such information is important for informing context-specific recommendations to improve screening levels in areas where they may be low.

Another limitation of this study is that the survey questions did not provide information about the number of times the women had been tested, or if a woman was tested before the recommended age. Experts who have participated on WHO missions to some of these countries noticed that over-screening (once a year or up to five times a year according to local professionals) take place in many eastern European and Central Asian countries. In those countries, the perception of the necessity of having a smear test once a year, inherited from Soviet times, still persists among health providers and women. Over testing can lead to false positives, false negatives, psychosocial concerns, over-diagnosis, over-treatment), without amplifying the benefit (number of true precancers and cancers detected), in addition to, wasted resources (7,20). Future research is needed to measure more precisely the magnitude of over screening issues in Central Asian and Eastern European countries. At the same time, it should be noted that large proportion of women in low resource settings have not been tested at all. In those settings, increasing screening coverage is a priority.

Additionally, future work could assess the woman’s age at the time of testing. In this study we included surveyed women aged 30-49 years who reported ever having a cervical cancer screening test. We chose this age range because WHO recommends that cervical cancer screening should be performed at least once for every woman in the target age group where the most benefit can be achieved (30-49 years)(4), and because of its link with the Global Monitoring Framework for NCDs(18). That said, the WHO recommended performance indicator for cervical cancer screening and treatment is the “percentage of women aged 30-49 years who report ever having had a cervical cancer test(18).” The way the indicator is defined left open the possibility that women who confirmed that they had been tested were tested at a young age (for example at age 20 years, and not since then), and not during the recommended age range of 30-49 years or according to national screening target ages. The WHO strategy for elimination of cervical cancer recommends that 70% of women be screened with a high performance test by age 35 years and again by 45 years (14), so having an indicator that collects information not only on whether a woman was tested for cervical cancer, but also how old she was, the number of tests she received, and whether or not this was part of a screening test or whether it was the response to clinical symptoms would be valuable. There is an important distinction between being “tested” for cervical cancer and being “screened” and future research must differentiate between these two activities. A refinement of the cervical cancer screening indicator is underway, taking into account new evidence(47). Future studies may benefit from the revised indicators.

Another consideration relates to the possible generational effects of HPV vaccination and coverage. There is variation across Europe in terms of availability of total or partial charge HPV vaccination, with higher levels of immunization in higher income countries in Western Europe(34). Currently, only a few Eastern European and Central Asian countries have HPV vaccination programmes (48), and they were introduced recently. It is too early to examine the generational effects in those countries, but HPV vaccination is a core component of the WHO Global Strategy for cervical cancer elimination(14).

Furthermore, this paper did not categorize or compare the countries by healthcare systems or whether the screening was organized or opportunistic. Evidence suggests that population-based cervical cancer screening programs offering smear tests every three to four years in women over 30 have substantially reduced cervical cancer incidence (19,49). Further information on presence of national screening programs, type of test used, targeted age ranges, coverage of the population and whether the program is population-based or opportunistic is currently being collected in the WHO Country Capacity Survey (CCS) for the Prevention and Control of Non-communicable Diseases(50).

Another limitation of this study is that we do not differentiate between the types of testing that women received (many of the women who were surveyed may not have known the precise methods of sample analysis, and in many countries they offer a mix of different types of staining), so it is important to interpret the findings with caution and to acknowledge that state of the art methods are not always employed. Future research is needed to address the barriers which block effective introduction of organized screening programs. Future work is also needed to examine how testing practices within countries compare with national-level policies and targets, in order to inform further exploration of possible policy bottlenecks and areas for implementation research. Qualitative research with policy makers, medical professionals and women may help to identify challenges and help inform questions about how countries should best use scarce resources, particularly in low-income settings where resource constraints may impede full implementation of the cervical cancer elimination strategy.

Furthermore, for cervical cancer prevention to be effective, women with positive screening test results must receive effective treatment (the “screen-and-treat” or “screen, diagnose and treat” approach), in order to reduce loss to follow-up or a time lag for women to receive treatment(4). This study assessed the first component of this approach (testing of the women), but it did not assess whether or not appropriate and timely follow-up measures were taken.

## CONCLUSIONS

In conclusion, this paper establishes the most recent data for lifetime cervical cancer smear testing levels for women aged 30-49 years across the WHO European Region. Findings indicate that levels of smear testing are lower in Eastern Europe and Central Asian countries compared to Western Europe, and that they were also lower among lower-income countries. Information on testing levels which are comparable between countries and groups, as well as over time, can play an important role in raising awareness and in motivating political leaders to take action. The findings here indicate that nearly all countries need to do more to improve their reach among women of lower socioeconomic status. These findings can be useful to advocate for better screening, particularly among low-income women and women living in Central Asia and Eastern Europe. We hope that this publication will generate additional research into the root causes of issues including over testing, poor coverage and low levels of follow-up. This paper looked at one of many aspects of cervical cancer control—smear testing of women as a first step in the screening process—but effective strategies toward cervical cancer elimination will also require coordinated implementation of population-based, quality-assured HPV vaccination programmes, screening programmes (with appropriate follow-up), quality management of invasive cervical cancer (including palliative care) and cancer registries.

## ACKNOWLEDGEMENTS

The writing group would like to gratefully acknowledge the support of the participating Member States, study participants, data collectors, researchers and study coordinators. The writing group expresses thanks to Dr Tatyana Migal, Dr Rosa Djakypova, Dr Ata Boppyev, Mr Safar Sayfuddinov, Dr Elena Fidarova and Dr Vitaly Smelov.

The writing group takes sole responsibility for the content of this article and the content of this article reflects the views of the authors only. JW, IR, MB, MC, BM, NB and JB are staff members of the WHO. The WHO is not liable for any use that may be made of the information contained therein.BB and TR are supported by the National Institute for Health Research (NIHR) Collaboration for Leadership in Applied Health Research and Care North West Coast (CLAHRC NWC), and the views expressed here are those of the authors alone.

1 Global Monitoring Framework on NCDs. Indicator 25: Cancer screening. World Health Organization https://www.who.int/nmh/ncd-tools/indicator25/en/ (accessed Feb 14, 2019).

2 Gakidou E, Nordhagen S, Obermeyer Z. Coverage of cervical cancer screening in 57 countries: low average levels and large inequalities. *PLoS medicine* 2008; **5**: e132.

3 Akinyemiju TF. Socio-economic and health access determinants of breast and cervical cancer screening in low-income countries: analysis of the World Health Survey. *PloS one* 2012; **7**: e48834.

4 Altobelli E, Rapacchietta L, Profeta VF, Fagnano R. HPV‐vaccination and cancer cervical screening in 53 WHO European Countries: An update on prevention programs according to income level. *Cancer medicine* 2019; **8**: 2524–34.

5 Van Minh H, My NTT, Jit M. Cervical cancer treatment costs and cost-effectiveness analysis of human papillomavirus vaccination in Vietnam: a PRIME modeling study. *BMC Health Services Research* 2017; **17**: 353.

6 Subramanian S, Trogdon J, Ekwueme DU, Gardner JG, Whitmire JT, Rao C. Cost of Cervical Cancer Treatment: Implications for Providing Coverage to Low-Income Women under the Medicaid Expansion for Cancer Care. *Womens Health Issues* 2010; **20**: 400–5.

7 Lortet-Tieulent J, Georges D, Bray F, Vaccarella S. Profiling global cancer incidence and mortality by socioeconomic development. *Int J Cancer* 2020; **147**: 3029–36.

8 World Health Organization. Incidence of cervix uteri cancer per 100,000. European Health Information Gateway. https://gateway.euro.who.int/en/indicators/hfa_377-2360-incidence-of-cervix-uteri-cancer-per-100-000/ (accessed July 30, 2019).

9 Bray F, Ferlay J, Soerjomataram I, Siegel RL, Torre LA, Jemal A. Global cancer statistics 2018: GLOBOCAN estimates of incidence and mortality worldwide for 36 cancers in 185 countries. *CA: a cancer journal for clinicians* 2018; **68**: 394–424.

10 WHO. Draft Global Strategy Towards Eliminating Cervical Cancer as a Public Health Problem. *WHO: Geneva, Switzerland* 2019. https://www.who.int/publications/m/item/draft-global-strategy-towards-eliminating-cervical-cancer-as-a-public-health-problem (accessed Oct 22, 2020).

11 Global Cancer Observatory from the International Agency for Research on Cancer and the World Health Organization. http://gco.iarc.fr/ (accessed July 30, 2019).

12 Comprehensive Cervical Cancer Control: A Guide to Essential Practice, 2nd edn. World Health Organization, 2014.

13 Aimagambetova G, Chan CK, Ukybassova T, *et al.* Cervical cancer screening and prevention in Kazakhstan and Central Asia. *Journal of Medical Screening* 2020; : 0969141320902482.

14 Znaor A, Ryzhov A, Corbex M, Pineros M, Bray F. Cervical cancer in the Newly Independent States of the former Soviet Union: incidence will remain high without action. *The Lancet* 2020; **(in press)**.

15 Markovic M, Kesic V, Topic L, Matejic B. Barriers to cervical cancer screening: A qualitative study with women in Serbia. *Social Science & Medicine* 2005; **61**: 2528–35.

16 WHO guidance note: comprehensive cervical cancer prevention and control: a healthier future for girls and women. World Health Organization, 2013 https://apps.who.int/iris/bitstream/handle/10665/78128/9789241505147_eng.pdf;jsessionid=7DD8F4CCF9FF9A5312A0DC0D69614131?sequence=3 (accessed Feb 14, 2019).

17 Chrysostomou A, Stylianou D, Constantinidou A, Kostrikis L. Cervical cancer screening programs in Europe: The transition towards HPV vaccination and population-based HPV testing. *Viruses* 2018; **10**: 729.

18 IARC Working Group on Evaluation of Cervical Cancer Screening Programmes. Screening for squamous cervical cancer: duration of low risk after negative results of cervical cytology and its implication for screening policies. *British Medical Journal (Clinical Research Edition)* 1986; : 659–64.

19 Santourian A, Kitromidou S. Quality report of the second wave of the European Health Interview survey. https://ec.europa.eu/eurostat/documents/7870049/8920155/KS-FT-18-003-EN-N.pdf/eb85522d-bd6d-460d-b830-4b2b49ac9b03 (accessed March 11, 2019.

20 Guide to cancer: early diagnosis. Geneva, Switzerland: World Health Organization, 2017.

21 WHO Regional Office for Europe. Screening programmes: a short guide. Increase effectiveness, maximize benefits and minimize harm. Copenhagen, 2020 https://apps.who.int/iris/bitstream/handle/10665/330829/9789289054782-eng.pdf.

22 WHO. To eliminate cervical cancer in the next 100 years, implementing an effective strategy is critical. 2020; published online Feb 4. https://www.who.int/news/item/04-02-2020-to-eliminate-cervical-cancer-in-the-next-100-years (accessed Oct 22, 2020).

23 World Health Organization. Major milestone reached as 100 countries have introduced HPV vaccine into national schedule. https://www.who.int/news/item/31-10-2019-major-milestone-reached-as-100-countries-have-introduced-hpv-vaccine-into-national-schedule (accessed Nov 5, 2020).

24 Kesic V, Poljak M, Rogovskaya S. Cervical cancer burden and prevention activities in Europe. *Cancer Epidemiology and Prevention Biomarkers* 2012; **21**: 1423–33.

25 Arbyn M, Rebolj M, De Kok IM, *et al.* The challenges of organising cervical screening programmes in the 15 old member states of the European Union. *European Journal of Cancer* 2009; **45**: 2671–8.

26 WHO. Assessing national capacity for the prevention and control of noncommunicable diseases: report of the 2019 global survey. 2020 https://www.who.int/publications/i/item/ncd-ccs-2019 (accessed Oct 12, 2020).
