# Supplementary material for: Cervical cancer testing among women aged 30–49 years in the WHO European Region
Source: Eur J Public Health. 2021 Sep 7;31(4):884–9. doi: 10.1093/eurpub/ckab100 (PMC8514175; doi:10.1093/eurpub/ckab100)
Supplement: ckab100_Supplementary_Data [file ckab100_supplementary_data.zip › ckab100-suppl_data/ejph-2020-07-om-0880-File006.docx]

**Supplementary References**

41. World Health Organization. Major milestone reached as 100 countries have introduced HPV vaccine into national schedule [Internet]. [cited 2020 Nov 5]. Available from: https://www.who.int/news/item/31-10-2019-major-milestone-reached-as-100-countries-have-introduced-hpv-vaccine-into-national-schedule

42. WHO. To eliminate cervical cancer in the next 100 years, implementing an effective strategy is critical [Internet]. 2020 [cited 2020 Oct 22]. Available from: https://www.who.int/news/item/04-02-2020-to-eliminate-cervical-cancer-in-the-next-100-years

43. Bray F, Ferlay J, Soerjomataram I, Siegel RL, Torre LA, Jemal A. Global cancer statistics 2018: GLOBOCAN estimates of incidence and mortality worldwide for 36 cancers in 185 countries. CA Cancer J Clin. 2018;68(6):394–424.

44. Markovic M, Kesic V, Topic L, Matejic B. Barriers to cervical cancer screening: A qualitative study with women in Serbia. Soc Sci Med. 2005 Dec 1;61(12):2528–35.

45. Chrysostomou A, Stylianou D, Constantinidou A, Kostrikis L. Cervical cancer screening programs in Europe: The transition towards HPV vaccination and population-based HPV testing. Viruses. 2018;10(12):729.

46. IARC Working Group on Evaluation of Cervical Cancer Screening Programmes. Screening for squamous cervical cancer: duration of low risk after negative results of cervical cytology and its implication for screening policies. Br Med J (Clin Res Ed). 1986;659–64.
